# Microarray-Based Methodology for Lipid Profiling, Enzymatic Activity, And Binding Assays in Printed Lipid Raft Membranes from Astrocytes and Neurons

**DOI:** 10.1021/acs.analchem.4c02421

**Published:** 2024-12-24

**Authors:** Laura Sánchez-Sánchez, Roberto Fernández, Egoitz Astigarraga, Gabriel Barreda-Gómez, María Dolores Ganfornina

**Affiliations:** † IMG Pharma Biotech S.L, Zamudio 48170, Spain; ‡ Instituto de Biomedicina y Genética Molecular, Unidad de Excelencia, University of Valladolid-CSIC, Valladolid 47003, Spain

## Abstract

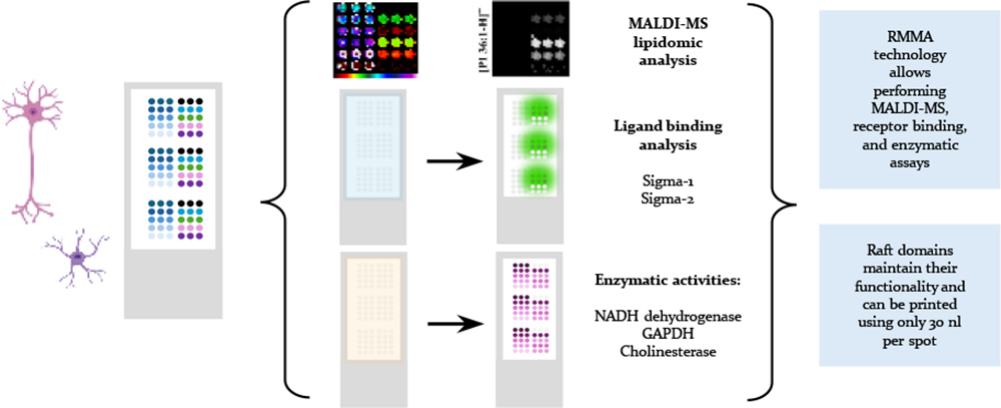

Lipid rafts are liquid-ordered domains in which specific enzymes and receptors are located. These membrane platforms play crucial roles in a variety of signaling pathways. Alterations in the lipid environment, such as those elicited by oxidative stress, can lead to important functional disruptions in membrane proteins. Cell membrane microarrays have emerged in the past decade as a powerful methodology for the study of both lipids and membrane proteins at large scales. Based on that technology and the importance of liquid-ordered subdomains, we have developed a new printed lipid raft technology with a preserved native protein structure and lipid environment. To validate this technology and evaluate its potential for different aims, raft membrane microarrays (RMMAs) containing two different cell types (astrocytes and neurons) and three different conditions (astrocytes in control situation, metabolic stress, and oxidative stress) were developed. To study differences in lipid profiles between raft domains, the MALDI-MS assay was performed on RMMAs. To evaluate the preservation of native protein activities (enzymatic activity and ligand binding) in the printed raft domains, differences in NADH oxidoreductase, GAPDH, cholinesterase activities, and sigma-1 and sigma-2 binding assays were performed. We demonstrate the performance of this new microarray technology, adapted to membrane subdomains, as valid to explore changes in lipid composition and protein activities in raft domains from brain cell lines under different stress conditions relevant for neuropathology.

## Introduction

Particular combinations of glycerophospholipids (GPs), sphingolipids (SLs), glycerolipids (GLs), and sterols (STs) constitute the complete lipidome that can be organized in liquid ordered and disordered domains within membranes.^1^ Raft microdomains, also known as lipid rafts, are heterogeneous and metastable^2^ liquid ordered domains with a dynamic structure^3^ that are enriched in SLs and cholesterol.^4^ Their main characteristic apart from their composition is their resistance to detergent disruption.^5^ These domains are present in the external part of the plasma membrane leaflet^6^ and could be found in mammalian cell endosomes.^5,7^ Additionally, raft-like domains have been observed in various organelles such as the endoplasmic reticulum (ER), lysosomes, or mitochondria.^8−10^

In addition to their existence in a variety of cell locations, the protein compositions of raft domains can change due to exposure to different stimuli. Examples of proteins found in these domains are the different complexes of the oxidative phosphorylation system,^11^ different brain receptors, such as sigma-1 and sigma-2, or flotillin-1, a protein enriched in raft domains and thus considered a general raft biomarker.^12,13^ Moreover, acetylcholinesterase (AChE),^14^ a protein that performs the hydrolysis of the neurotransmitter acetylcholine, can be in raft due to its glycosylphosphatidylinositol (GPI) anchor.^15,16^ Sigma domains are nonopioid receptor brain proteins implicated in certain psychiatric disorders^17^ that have also been suggested to participate in neurodegenerative diseases, such as Alzheimer disease.^18^ These receptors have been found in the mitochondria-associated endoplasmic reticulum membrane (MAM), specifically in detergent-resistant microdomains.^19^

Changes in the organization or protein composition of a lipid raft can alter the membrane’s local environment and entail disturbances over cellular functionality, which is particularly relevant in the nervous system where altered neurotransmission or protein clustering clearly contributes to neuropathological events.^20,21^ High oxidative damage have been reported in pathologies such as Alzheimer disease and closely correlates with amyloid and tau pathologies.^22^ Oxidative damage produced by reactive oxygen species is predicted to produce changes in membrane rafts in pathologic conditions, such as lipid peroxidation^23^ or raft disruption.^24^ Reactive oxygen species can be produced by different enzymes and can be triggered by exposition to serum starvation^25^ or to a pro-oxidant agents like paraquat.^26^ Therefore, as raft lipidome changes are expected in oxidative stress conditions, the analysis of lipid fingerprints in raft domains from different brain cell types exposed to metabolic and oxidative stress conditions is of special interest. Nevertheless, the size of lipid raft domains is a limiting factor for several techniques, such as HPLC-MS analysis, which requires not only high amounts of samples but also much time for data acquisition.^27,28^ On the other hand, while MALDI requires less quantity, when the sample is spotted with a standard method, requiring generally less than 1 μL, it has the limitation that the sample deposition is usually nonhomogeneous.^29^

Cell membrane microarray technology has been successfully applied to the analysis of lipid fingerprint in samples from cell cultures and tissues homogenates.^30^ In this work, we expand this technology for the analysis of printed raft domains. Making the microarray technology compatible with lipid raft analysis should represent a relevant improvement, allowing the analysis of many and varied raft domain samples in the same platform, with small amounts of sample required. For this purpose, the method of separation of lipid rafts, generally by detergents, needs to be adapted to ensure compatibility with the printing method, MALDI-MS, and enzymatic assays techniques.^31,32^ In this work, we perform and validate a novel methodology for raft purification and raft printing using microarray technology, maintaining the functionality and making them compatible with MS, enzymatic, and binding assays. We apply this new methodology to cell types and conditions relevant for neuropathology.

## Experimental Section

### Cell Lines and Culture Conditions

Astrocytoma 1321N1 and neuroblastoma SH-SY5Y cell lines were purchased from ECACC (ECACC-86030402) and ATCC (ATCC CRL-266), respectively. Both cell lines were cultured at 37 °C in a humidity-saturated atmosphere containing 5% CO_2_ using a cell incubator (Hera Cell 150, Hareaus, Hanau, Germany). Three different culture conditions were used: control situation and low serum starvation with and without paraquat exposure.

#### Control Situation

The 1321N1 cell line was cultured using DMEM medium 1 g/L glucose supplemented with 10% heat-inactivated fetal bovine serum (FBS), 1% l-glutamine (l-Glut), and 1% penicillin/streptomycin (P/S). The SH-SY5Y cell line was cultured using 1:1 DMEM:F12 medium supplemented with 10% heat-inactivated FBS, 1% nonessential amino acids, and 1% P/S.

#### Low Serum Starvation

Confluent 1321N1 cultures (around 10^5^ cells/cm^2^) were switched from the control medium to DMEM 1 g/L glucose, 0.2% charcoal-treated FBS, 1% l-Glut, and 1% P/S. Cells were harvested after 24 h of treatment.

#### Low Serum Starvation with Paraquat Treatment

Confluent 1321N1 cultures (around 10^5^ cells/cm^2^) were switched from the control medium to low serum medium with 0.5 mM paraquat. Cells were harvested after 24 h of treatment. For simplicity, this condition is labeled as “paraquat” in figures and legends.

### Membrane Extraction for Lipid Raft Purification

For membrane purification, around 10^6^ cells were used as described.^9,33^ Cell pellets (1321N1 or SH-SY5Y) were resuspended in 5 mL of TNE buffer (Tris-HCl 50 mM, NaCl 150 mM, EDTA 5 mM, pH 7.4) with protease inhibitors (PrI) and were homogenized in a Potter S system (Satorius AG, Göttingen, Germany), with 20 strokes rotating at 400 rpm. Supernatants were collected, transferred to quick-seal 5.6 mL centrifuge tubes (ref 363963, Beckman Coulter, Brea, CA, USA), and centrifuged at 100000 *g* in an optimal 100 XP ultracentrifuge (Beckman coulter, Brea, CA, USA, 100Ti rotor) for 75 min. Supernatants were discarded, and membrane pellets were resuspended in 200 μL of TNE with PrI. Protein quantity was determined using a Pierce MicroBCA protein assay (ref 23235, ThermoFisher Sci., Waltham, MA, USA).

### Detergent-Free Method for Lipid Raft Isolation

#### Sonication Raft Separation

Membrane preparations (300 μg of proteins) were resuspended in 2 mL of TNE with PrI. The suspension was sonicated (Vibracell 75115, ThermoFisher Bioblock scientific S.L, Waltham, MA, USA) 5 times with 30% amplitude using intervals of 20 s with 1 min cooldown periods between each sonication pulse using a TipCB33-3363658 tip (BochemLabordebarf, Weilburg, Germany). Immediately after the sonication, each sample was mixed with 4.4 mL of 80% sucrose prepared as described below.

### Sucrose Step-Gradient Preparation

For the 80% sucrose solution, sucrose was dissolved in TNE (by heating at 60 °C, avoiding overheating and caramelization), and PrI were added after cooling. Serial dilutions of the 80% solution are performed in TNE with PI to make 35%, and 5% sucrose solutions. Sonicated membranes (1 mL) are mixed with 80% sucrose to get a final concentration of 55% and placed at the bottom of a 12.5 mL ultracentrifuge tube (ref 344060, Beckman Coulter, Brea, CA, USA). A 6 mL layer of 35% sucrose is placed with care on top of the sample, and a final 3 mL layer of 5% sucrose is on top.

#### Raft Separation by Ultracentrifugation

Sucrose gradients were centrifuged at 4 °C and 102000 *g* (Beckman coulter optimal 100 XP ultracentrifuge, SW40 rotor, Brea, CA, USA) for 24 h. After ultracentrifugation, tubes are always kept on ice, and the raft domains (present in the interphase between 5% and 35% sucrose) are collected in a 1 mL fraction. To check for the presence of raft domains, 300 μL of each fraction in the gradient was precipitated (see below) and analyzed by Western blot assay. The rest of the volume (700 μL) was diluted up to 5.6 mL with TNE with PI to dilute the excess sucrose. After gentle mixing, they were ultracentrifuged in quick-seal centrifuge tubes as described above for 2.5 h to ensure the correct precipitation of the raft pellet. Supernatants were discarded, and pellets were resuspended in 100 μL of TNE with PI, transferred to 1.5 mL Eppendorf tubes, and centrifuged again at 4 °C (6 h, 16100 *g* in 5415R microcentrifuge, Eppendorf, Hamburg, Germany). After the supernatants were discarded, raft samples were stored as pellets at −80 °C until usage.

### Trichloroacetic Acid Protein Precipitation and Western Blot Assay

For Western Blot analysis, an adaptation of the protocol used by Corraliza-Gomez was used.^9^ For more details, see the Supporting Information file.

### Raft Membrane Microarrays (RMMAs) Printing Method

Raft pellets were defrosted on ice for 15 min, resuspended in printing buffer, adjusted to a concentration of 5 mg/mL, and incubated overnight at 4 °C. Raft preparations were printed onto preactivated glass slides using a noncontact microarrayer with a solenoid tip (Nanoplotter NP2.1, GeSiM Bioinstruments, and Microfluidics, Radeberg, Germany), placing 3 replicates of each sample (3 nl/drop, 10 drops/spot). Different concentrations of rat brain cortex whole membranes were used as the inner control, and the printing buffer was used as the negative control. Preactivation of the glass slides was carried out as previously described.^34^ Printing was carried out under controlled humidity (relative humidity 60%) at a controlled temperature of 4 °C. Distance between spots was set at 950 μm. To ensure the correct spot adherence, RMMAs were let dry at 4 °C and 60% relative humidity for 45 min before being stored at −20 °C until usage.

### Protein Quantification by Bradford Staining in RMMAs

After at least 2 days at −20 °C, Bradford staining is performed in RMMAs to test correct membrane adherence and analyze the protein concentration of each spot after printing. RMMAs were defrosted inside a desiccator chamber for 1 h. Afterward, RMMAs are completely immersed in Bradford staining (4.7% Coomassie blue, 8.5% *ortho*-phosphoric acid) at 4 °C for 1 h in darkness. After being dipped in distilled H_2_O at room temperature, stained RMMAs were dried out with a small fan. Color signal was acquired with an Epson V750 pro canner (Seiko Epson Corporation, Suwa, Nagano, Japan), and digital images were analyzed and quantified using ImageScanner Software (IMG Pharma S.L, Zamudio, Spain). Protein total quantity in each spot is determined using the known concentrations of rat brain cortex spots as a standard curve.

### MALDI-MS in RMMAs

To perform lipidomic analysis, RMMAs were coated with a uniform film of approximately 0.2 mg/cm^2^ with the aid of a standard glass sublimator (Ace Glass 8233, Vineland, NJ, USA). 1,5-Diaminophtalene (DAN) and 2-mercaptobenzothiazole (MBT) were used for negative and positive ion-modes, respectively. The matrixes were sublimated for 19 and 17 min, respectively. RMMAs were then scanned. Since in MALDI imaging experiments the coverage, pixel quantity, and resolution of each experiment depend on the spot’s diameter, the separation between spots and the quality of the samples were analyzed. Since each spot has a diameter of 450 μm, the array area was explored following coordinates in a grid with 150 μm between-nodes separation. We used an LTQ-Orbitrap XL mass spectrometer (Thermo Fisher Scientific, Waltham, Massachusetts, USA) equipped with a MALDI source with a N_2_ laser (60 Hz, 100 μJ/pulse maximum power output). The laser spot is an ellipsoid of approximately 50–60 μm × 140–160 μm. Two microscans of 10 shots/pixel were used, with laser power outputs of 30 and 20 μJ for MS– and MS+, respectively, and a resolution of 150 μm. Data loading included spectra normalization by total ion current (TIC), spectra alignment, and peak picking, filtering all the *m*/*z* values with intensity <0.5% of the strongest peak in the spectrum. Lipid *m*/*z* values and their annotations are listed in Table S1.

### Mitochondrial Electron Transport Chain Enzymatic Activities

To measure the activities of different complexes of the mitochondrial electron transport chain, the following assays were performed.

#### NADH-Oxidoreductase Activity Assay

RMMAs were placed in a desiccator for 30 min to ensure correct defrosting, and the area of each microarray was delimited with a hydrophobic barrier pen. RMMAs were incubated with a reaction solution (0.35 mM NADH, 0.1 mg/mL NBT, 50 μM decylubiquinone (dUQ), phosphate buffer (PB) 10 mM, pH 7.4) with and without sodium azide (10 mM) inside a humidity chamber at a controlled temperature of 24 °C for 4 h. The reaction was stopped by removing the reaction solution, gently washing the RMMAs in distilled water, and drying them at room temperature with a small fan.

#### Gliceraldehydo-3-phosphate Dehydrogenase Activity Assay

RMMAs were correctly defrosted, and the microarray area was delimited as indicated above. RMMAs were incubated with a reaction solution (20 mM glyceraldehyde-3-phosphate, 0.1 mg/mL NBT, 50 μM dUQ, cytochrome c 0.01%, PB 10 mM, pH 7.4). The reaction was stopped as described above.

### Cholinesterase Activity Assay

To study the cholinesterase, acetylcholinesterase, and butirylcholinesterase activities, the following protocols were performed.

#### Total Cholinesterase Activity Assay

RMMAs prepared as above were washed twice using Tris-maleate buffer (0.1 M Tris-maleate, pH 6). RMMAs were incubated with a reaction solution (65 mM Tris-maleate pH 6, 5 mM sodium citrate, 3 mM copper sulfate, and 5 mM potassium ferricyanide with and without 0.74 mg/mL acetylcholine iodide). The reaction mixture was incubated for 16 h in humidity chamber in darkness. The reaction was stopped by cautiously removing the reaction solution and washing twice with Tris-maleate buffer for 10 min. RMMAs were then dipped into distilled water and dried as explained above.

#### Butirylcholinesterase Activity Assay

RMMAs prepared as described above were washed twice using Tris-maleate buffer and incubated with the same reaction solution as in total cholinesterase assay but with BW284 (1 mM) added as a selective inhibitor for acetylcholinesterase activity. After 16 h incubation in a humidity chamber in darkness, the reaction was stopped and the RMMAs were dried as explained above.

To obtain the acetylcholinesterase activity, the difference between the butirylcholinesterase and total cholinesterase activities was calculated.

### Sigma-1 Receptor Ligand-Binding Assay in RMMAs

To analyze whether the RMMAs are suitable for binding assays, sigma-1 and sigma-2 receptor binding assays were performed. First, RMMAs were defrosted for 60 min at room temperature inside a desiccator chamber. A preincubation was performed in washing buffer (50 mM Tris-HCl, pH 7.4, 1 mg/mL bovine serum albumin (BSA), 1% sodium deoxycolate) at 24 °C inside a water bath for 60 min. RMMAs were then incubated for 150 min at 37 °C inside a water bath with washing buffer with sigma receptor fluorescent ligand CELT-483 (50 nM) in the presence or absence of haloperidol (10 μM), as a receptor antagonist, or L6 as a selective sigma-1 masking agent. Later RMMAs were washed gently in distilled water at 4 °C and fixed in 4% paraformaldehyde for 30 min at room temperature. The fixed microarrays were washed with PBS-TTD (PBS, 0.5% Tween-20, 0.5% Triton X-100, 1% sodium deoxycholate) and dried with a cold air current at 4 °C in darkness. Signal acquisition was performed using a ChemiDoc MP Imaging System with red illumination and a 695/55 filter. Images obtained were analyzed and quantified using Image Lab software (Bio-Rad, Hercules, CA, USA).

### Statistical Analysis of MS Data

Statistical analysis was performed using Graphpad Prism Software from Dotmatics (Boston, Massachussets, USA). α was set to 0.05 in all tests.

#### Outlier Detection

For possible outlier spectrum detection, a two-tailed Pearson correlation test was performed for each ionization mode separately. A correlation of less than 0.7 between replicates is considered as an outlier value and discarded.

#### Monovariable Analysis

To test the normality of each variable, a Saphiro–Wilk normality test was performed. To elucidate if there was any statistically significant difference in lipid relative abundance between the different spectra obtained, the Mann–Whitney rank nonparametric test was performed for every comparison.

### Pixel Cluster

Segmentation of the pixels in the array image was done using a modified version of the segmentation algorithm RankCompete based on the properties of Markov chains^35^ to define random walkers^36,37^ competing to divide the imaging experiment into two segments. However, by definition, the RankCompete algorithm divides the experiment into two segments, and our array data may contain a variable number of segments. Therefore, we used a variation of the Divisive Analysis algorithm (DIANA) to create a variable number of walkers. Thus, the final software is a segmentation algorithm based on DIANA and uses RankCompete as a split function. Once the segments were obtained, correlations between them were calculated, and the value was used to assign a color to each segment using a color scale and 1-correlation between the segments. In this way, the two segments that present the lowest correlation occupy the two extremes of the scale, and those segments with more similar average spectra receive colors that are closer to each other in the scale.

### Multivariable Analysis

Different methods were used for analysis and classification, divided into unsupervised and supervised methods. For the principal component analysis, normalized data were used, whereas for the other methods data sets with a reduced number of variables after principal components analysis were used instead.

#### Principal Component Analysis

Principle component analysis is a suitable method to describe the behavior of the samples. To reduce the number of samples, we selected the 50 best-ranked variables using the ANOVA test. Afterward, the number of principal components (PC) that can explain more than 90% of the variability were selected, with a % variability explained larger than 0.5% in each additional PC.

#### K-Nearest Neighbors’ Classification Method

The K-nearest neighbors classification method is a supervised method in which each data point is classified to the class that is most prevalent out of the closest points. The K number of neighbors was set to 5 using The Euclidean distance and uniform weight.

#### Naïve Bayes

Naïve Bayes is a supervised method based on the conditional probability theorem of Bayes.

#### Random Forest

The random forest supervised classificatory method was based on decision trees. The number of decision trees was set to 10, and subsets with less than 5 features do not split into new decision trees.

#### Neural Networks

The neural network method is a supervised method composed of different layers of “neurons”. The number of neurons per hidden layer was set at 200, with a maximum of 350 iterations. The activation function used was a rectified linear unit function (ReLu; eq 1).
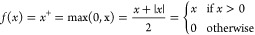
1

The solver for the weight optimization used was a stochastic gradient descent.

For all the supervised methods, the validation was performed using cross-validation of 10-fold. All the analyses were performed using Orange^38^ open software tools from the University of Ljubljana (Ljubljana, Slovenia).

### Signal Acquisition for Enzymatic Assays, Data Processing, And Statistical Analysis

For all the activity assays, the color signal was acquired with an Epson V750 pro-scanner, and digital images were analyzed and quantified using the software ImageScanner (IMG Pharm Biotech S.L, Spain). Data obtained were normalized with respect to the total protein quantity obtained by Bradford assay in the RMMA. Data handling and analysis were carried out using Excel and Graphpad software (version 9.2). The identification of outliers was carried out using the following equations (eq 2 and 3):
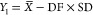
2
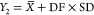
3where DF is the deviation factor, SD is the standard deviation, and *X̅* is the mean

Points lower than *Y*_1_ or higher than *Y*_2_ were identified as outliers. For the analysis, a deviation factor of 1.25 was used, and data were expressed as means of independent data points ± SD. The results were analyzed using one-way two-tailed ANOVA with Tukey’s posthoc test, and α was set as 0.05.

## Results

### Lipid Raft Preparation

**Figure 1:**
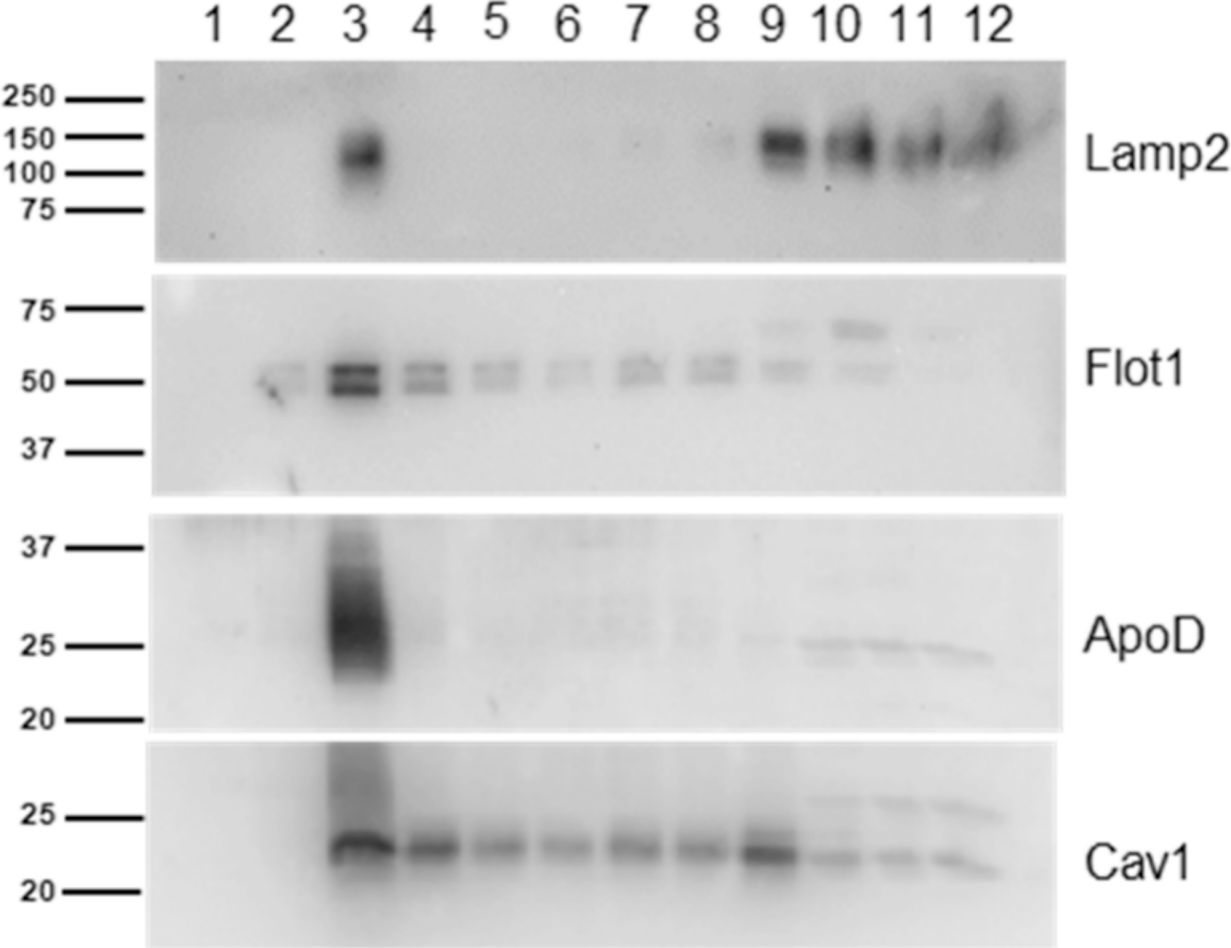
Western blot assay with markers for both raft and nonraft membrane subdomains. Example for raft preparation of 1321N1 cells in a control situation. Note that part of the strong ApoD signal was still present after stripping and is visible above the Cav1 signal.

Western blot assay was performed in all of the fractions obtained after the step-gradient ultracentrifugation in order to check the correct separation of the membrane subdomains. Only fraction 3, positive for Lamp2, Flotillin1, Apolipoprotein, and Caveolin1,^10^ was chosen as the lipid raft-enriched fraction and was used for printing RMMAs in every condition and cell type. Nonraft membranes remain at the bottom of the gradient and are characterized by high presence of Lamp2 and only minor traces of the other three markers (Figure 1).

The protein content was quantified for each sample, and the pellets were resuspended in printing buffer to reach a concentration of 5 mg/mL of protein.

### Lipid Profiling

**Figure 2:**
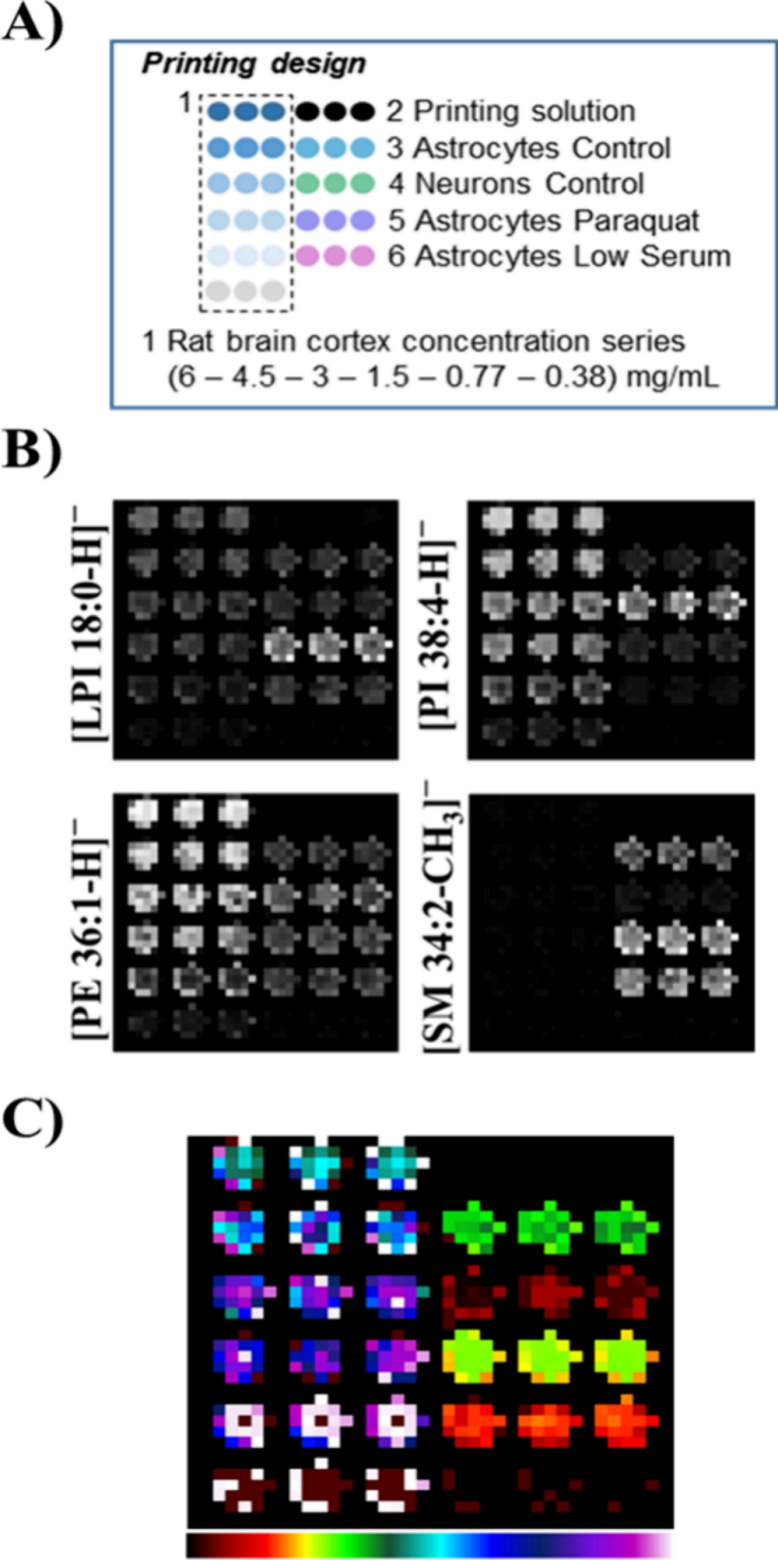
Lipid fingerprint analysis in RMMAs from astrocytic and neuronal cell lines using mass spectrometry in MS–. (A) Printing design for microarrays. Standard curves and samples were printed in triplicate. (B) Example images showing the relative abundance of selected lipids in raft samples or standards using gray scale. (C) Segmentation analysis using our modified RankCompete algorithm. Colors were assigned by using the rainbow color scale.

To demonstrate the capabilities of this methodology, microarrays containing lipid raft domains from human neuronal (control situation) and astrocytic cell lines (control situation and low serum starvation with or without paraquat exposure) were developed. Along with the lipid raft samples, the microarray also included membranes of rat brain cortex at 6 different concentrations as a quantitative standard curve. A line containing the printing solution was included as blank (Figure 2A). Each microarray spot contains 30 nL of a 5 mg/mL sample solution or different known concentrations for the rat brain cortex standards.

Following the procedure described in the “MALDI-MS in RMMAs” section, between 9 and 25 spectra (one per pixel) are obtained for each spot of the microarray. In order to get the average spectrum of each spot, a divisive hierarchical clustering is performed to segment the spectra in the spot (*R* > 0.9 between all the spectra in each segment), the more intense of which is identified and assigned as the average spectra of the sample (30). Those lipid average spectra were normalized to their total ion current (Figure S3) to compare the fingerprint changes over both cell lines and conditions. Immobilized lipid raft preparation showed clear source-dependent differences in the intensity of the different lipids analyzed by MALDI imaging, while no differences were observed between the replicates of a given sample. Our method is able to detect different distributions of certain lipids along the different cell types or conditions (Figures 2B and S1).

Spectra obtained in the MS– mode revealed that lipid raft preparations from the neuronal cell line are clearly different from those of astrocytes. Figure 2B shows how the peak at 885.5499 ([PI 38:4 – H]^−^) reached higher relative intensity than other selected lipids only in neuronal rafts. The peak that corresponds to [LPI 18:0 – H]^−^ presented a higher relative intensity in rafts from astrocytic samples exposed to paraquat, whereas [PE 36:1 – H]^−^ (*m*/*z* = 744.5549) showed a similar relative intensity in all astrocytic samples regardless of treatment. As another example, [SM 34:2;O2– CH_3_]^−^ was present only in rafts from astrocytic samples and displayed a relative increase in intensity with both treatments. Surprisingly, this lipid exhibited a very low relative intensity in neuronal rafts and was absent in rat brain cortex membranes (Figure 2B). To test for intraexperiment reproducibility, hierarchical clustering with the HDC-RC segmentation algorithm was performed directly over the TIC-normalized data. Pixels with the same color have more similarity between their spectra than those with different colors. In this sense, replicates of each raft sample presented similar colors, which can be an indicative of good intraexperimental reproducibility (Figure 2C). Along with this analysis, lipid raft samples from both cell lines in different conditions presented different spectra, which make them distinguishable by their lipid fingerprint (Figure 2C). In general, more homogeneous results have been obtained in our cluster analysis from negative ion mode than from positive ion mode (compare Figure 2C with Figure S1C).

**Figure 3:**
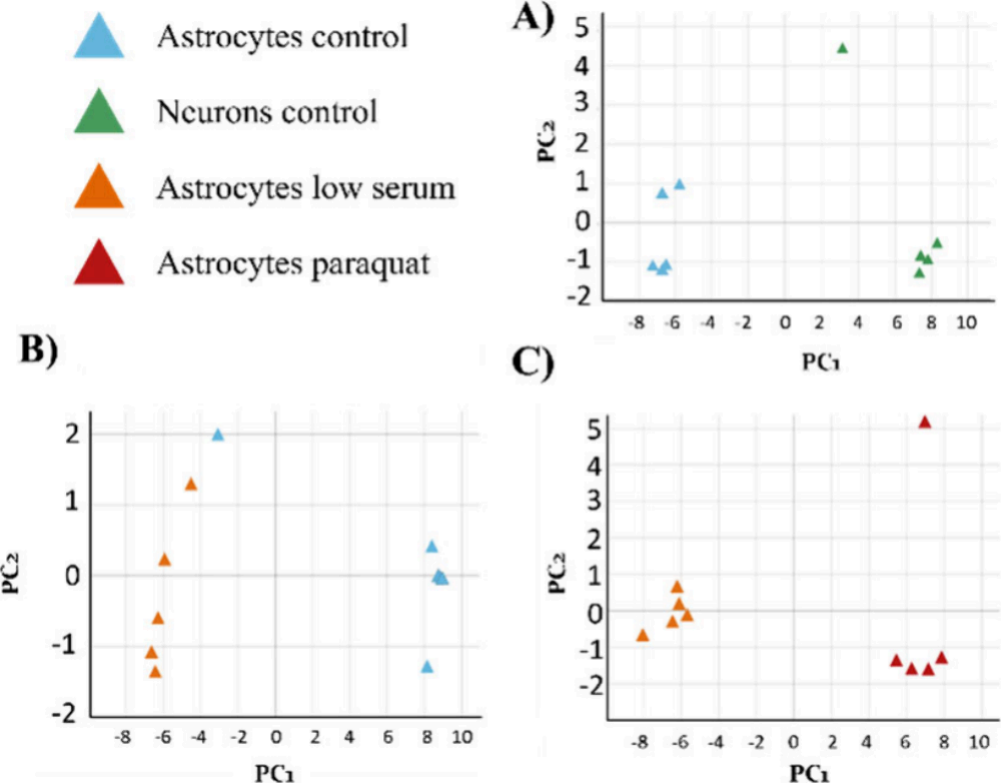
Principal component analysis (PCA) of lipid raft samples from neuronal and astrocytic cell lines under different conditions using MS– data. (A) PCA of astrocytic and neuronal rafts in control situations (92% variability explained by the first 2 principal components PC1 and PC2). (B) PCA of astrocytic rafts in metabolic stress and control situation (90% variance explained by 4 principal components PC1–PC4). (C) PCA of astrocytic rafts in metabolic and oxidative stress (98% variability explained by PC1 and PC2).

To answer the question of whether we can distinguish lipid rafts from different cell types or experimental conditions using their lipid fingerprints, we performed PCA and classification methods. The 50 best-ranked annotated lipids were used for PCA to compare rafts from both cell lines in the control situation (Figures 3A and S2A) and the astrocytic raft domains in control vs metabolic stress (Figures 3B and S2B) or in metabolic vs oxidative stress situations (Figures 3C and S2C). The selected lipids from MS– spectra were enough to ensure a complete separation between raft samples just in the first component when comparing neuronal with astrocytic rafts (Figure 3A) or the effect of oxidative stress in astrocytes (Figure 3C), and almost complete separation was caused by only metabolic stress (Figure 3B). With data obtained from MS+ spectra, the first principal component can separate groups in all comparisons (Figure S2).

In order to check if this lipid fingerprint can lead into a useful sample classification, different algorithms were used (kNN, neural network, Naïve Bayes and random forest), as explained in the Experimental Section. To this aim, we have performed the analysis with the whole lipid fingerprint and with the 50 best-ranked annotated lipids that presented higher differences among the conditions analyzed. In both cases when comparing metabolic stress with the control situation and oxidative stress against metabolic stress conditions, higher accuracy was obtained with the 50 best-ranked lipid analysis (Tables S2 and S3).

### Treatment and Cell Line Influence over the Lipidome of Printed Lipid Raft Domains

**Figure 4:**
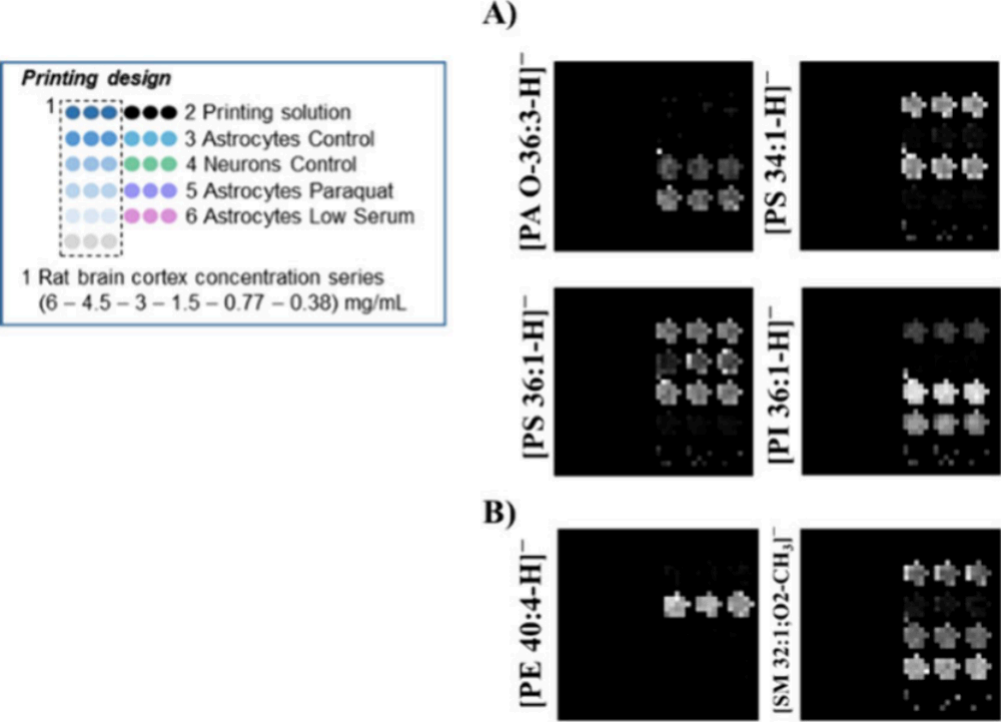
Relative abundance of selected example lipids in printed raft samples using the gray scale. Shown on the left is the design for the standard curve and samples, printed in triplicate. (A) Relative abundance of selected lipids that presented differences due to the treatment. (B) Relative abundance of selected lipids that presented differences between both cell lines in the control situation. No signal over the set threshold (0.1% of higher peak relative intensity) was detected in the rat cortex controls for these lipids.

Metabolic and oxidative stress conditions can be responsible for lipidome changes due to the presence of reactive oxygen species (Figure 4A). We have detected [PA O-36:3 – H]^−^ in astrocytic lipid rafts in both low serum conditions (with or without paraquat), showing a higher relative intensity in the last one. The opposite behavior was observed in [PS 36:1 – H]^−^ and [PS 34:1 – H]^−^, which displayed a higher relative intensity in astrocytic rafts under paraquat conditions and were barely present or even absent in neurons under control conditions. Similarly [PI 36:1 – H]^−^ was also highly present in paraquat conditions with low detection in the control or metabolic stress conditions (Figure 4A). In positive ion mode, differences in the relative abundance of [PC 34:1 + Cs]^+^ and [PC 35:2 + Cs]^+^ were found between conditions in astrocytic raft membranes, with higher relative intensity in the metabolic stress situation for [PC 35:2 + Cs]^+^ (Figure S1B). When studying differences between both cell lines in control situations (Figure 4B), [PE 40:4 – H]^−^ was, for example, only detected in rafts from the neuronal cell line, whereas [SM 32:1;O2 – CH_3_]^−^ showed a higher relative intensity in rafts from astrocytes in every condition tested and was absent in neuronal rafts. The same behavior was detected in positive-ion mode for [LPC 16:0 + Cs]^+^ (Figure S1B).

### Analysis of Enzymatic Activities in Printed Raft Domains

**Figure 5:**
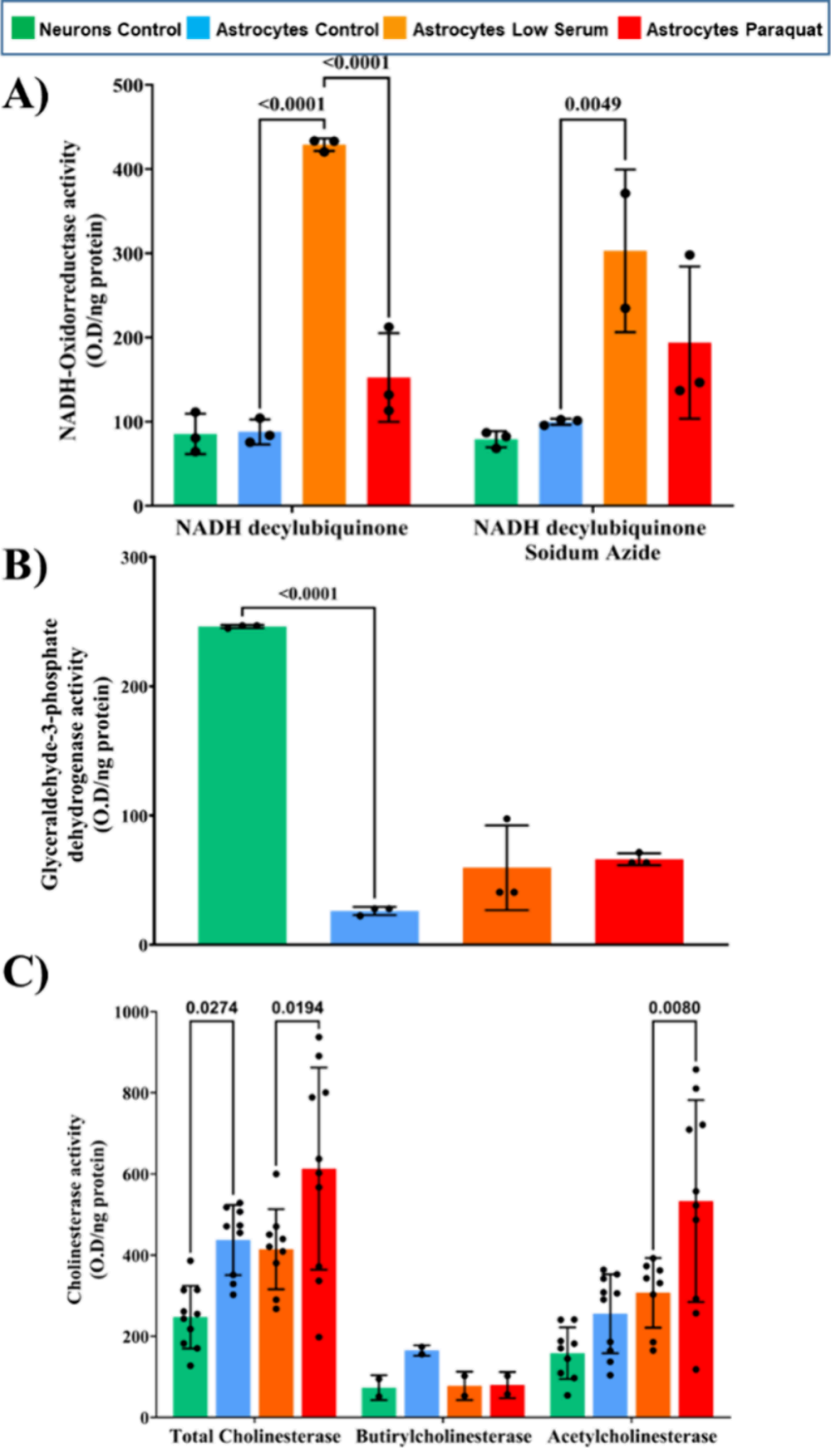
Enzymatic activity of selected enzymes in printed raft domains. (A) NADH oxidoreductase enzymatic activity in the presence or absence of a selective mitochondrial complex IV inhibitor. (B) Glyceraldehyde-3-phosphate dehydrogenase activity. (C) Total cholinesterase, butyrilcholinesterase, and acetylcholinesterase activity assay. Two-way ANOVA two-tailed test was performed in panels A and C. One-way ANOVA two-tailed test was used in panel B. In all cases, α was set at 0.05. Obtained *p*-values are shown in graphs. Data are expressed as mean ± SD.

Different enzymatic activities were analyzed in order to test the functionality of printed raft membranes. Lipid rafts contain a wide variety of integral and peripheral proteins that can be modified upon reaction to different stimuli such as the presence of metabolic or oxidative stress conditions. Moreover, it has been observed that different complexes of the oxidative phosphorylation system may exist in raft domains.^11^ To check these activities, a colorimetric assay was performed using NADH as the complex I substrate in the presence or absence of sodium azide, a selective inhibitor of complex IV (Figure 5A).

No differences were observed due to the presence of sodium azide under all conditions. Nevertheless, differences were observed in superoxide formation between the raft from astrocytes in low serum and control situations, which reached 429 ± 7 and 88 ± 15 O.D/ng immobilized protein, respectively (*p-*value <0.0001). The same tendency was observed between rafts from low serum starved astrocytes with and without paraquat, the last one achieving 153 ± 53 O.D/ng immobilized protein (*p-*value <0.0001) (Figure 5A). Moving forward to other enzymatic activities, GAPDH catalyzes the conversion of glyceraldehyde-3-phosphate to 1,3-biphosphoglycerate, an intermediate from the Krebs cycle, and is a key enzyme in the glycolytic pathway. It has been reported not only in the cytosolic fraction but also in membranes, either in rafts and nonraft domains.^39^ Significant differences were observed between rafts from neuronal (246 ± 1 O.D/ng immobilized protein) and astrocytic (26 ± 3 O.D/ng immobilized protein) cells in the control situation (*p-*value <0.0001). A slightly increasing tendency was observed due to oxidative stress when comparing raft domains from low serum starved astrocytes in the presence (60 ± 33 O.D/ng immobilized protein and absence (66 ± 5 O.D/ng immobilized protein) of paraquat exposure (Figure 5B).

Finally, we tested the activity of the ChE enzyme family that catalyzes the hydrolysis of choline esters. In particular, AChE catalyzes the hydrolysis of acetylcholine, while BuChE does so for butyrylcholine.^40^ In raft printed domains from neurons, ChE activity reached 247 ± 77 O.D/ng immobilized protein in comparison with 437 ± 86 O.D/ng immobilized protein determined in astrocytic rafts (*p*-value of 0.0274). Metabolic stress in astrocytes did not result in differences from the control situation, yielding 414 ± 99 O.D/ng immobilized protein. By contrast, the oxidative stress situation triggered by paraquat treatment resulted in increased activity in astrocytic rafts (613 ± 249 OD/ng immobilized protein) (*p-*value 0.0194). On the other side, BuChE activity, measured by using the BW284 AChE inhibitor, revealed that neuron printed rafts displayed about half the activity detected in printed rafts of control astrocytes, with 73 ± 3 and 165 ± 19 O.D/ng immobilized protein respectively. Control astrocytes also doubled the value of rafts for low serum starved cells in not only the absence but also the presence of paraquat, yielding in this case 78 ± 35 and 80 ± 32 O.D/ng immobilized protein, respectively. Regarding AChE activity, neuronal printed rafts received a color signal of 158 ± 64 O.D/ng immobilized protein and presented a lower activity than astrocytic rafts (256 ± 97 O.D/ng immobilized protein). No differences were observed between these control rafts and low serum starvation ones (307 ± 86 O.D/ng immobilized protein). However, a higher activity of AChE was obtained in paraquat-treated rafts (533 ± 249 O.D/ng immobilized protein), as happened with the total ChE activity (Figure 5C) (*p-*value 0.008).

### Performing Ligand-Binding Assays in Printed RMMAs: Sigma Receptors

**Figure 6:**
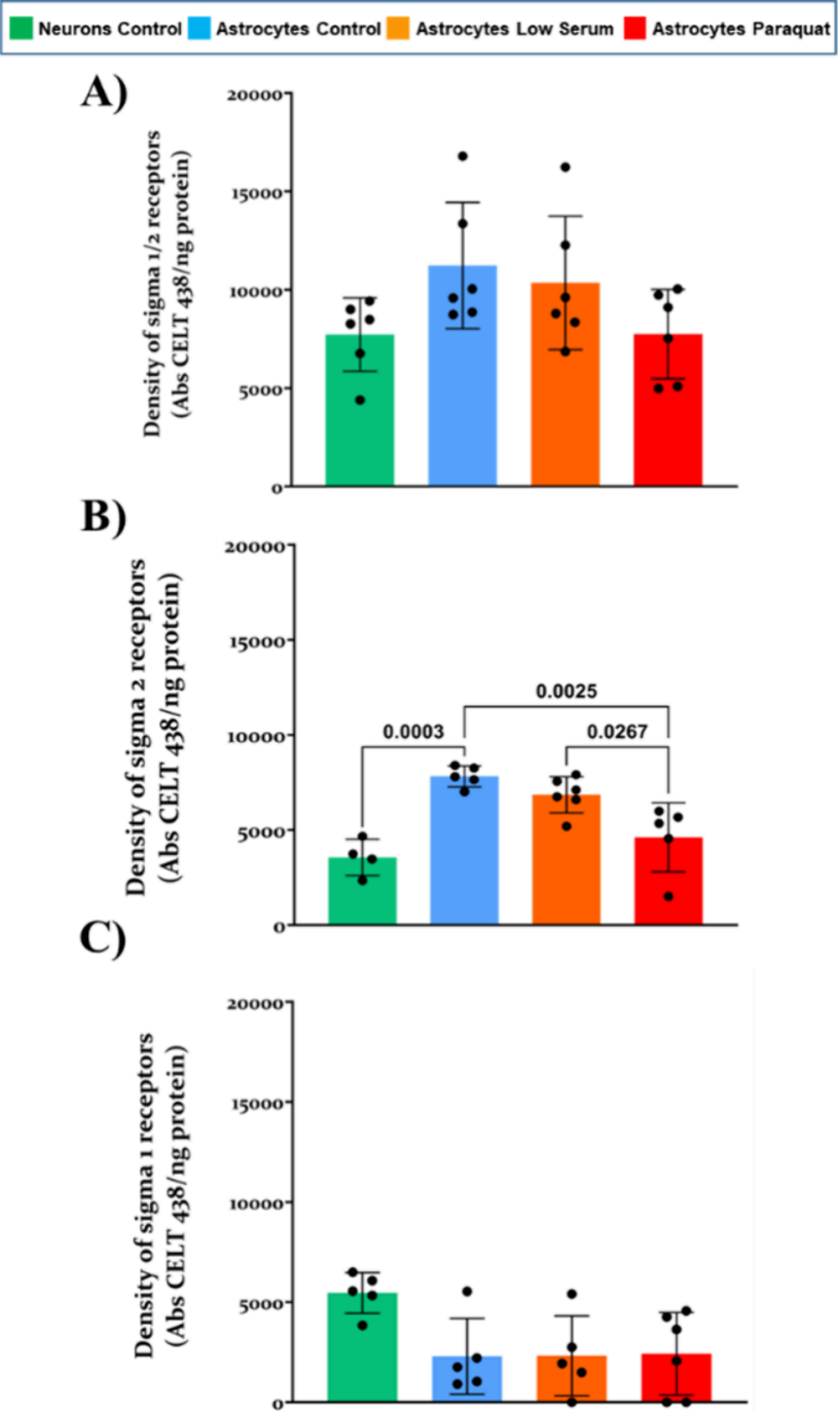
Fluorescent ligand binding assay for sigma-1 and sigma-2 receptors in printed lipid raft domains in different conditions. (A) Density of active total sigma-1 and sigma-2 receptors. (B) Density of sigma-2 receptors using a sigma-1 masking agent. (C) Density of sigma-1 receptors calculated as de difference between panels A and B. One-way ANOVA two-tailed wtest ith Tukey’s posthoc was carried out with α set at 0.05, obtained *p-*values are shown in graphs. Data are expressed as mean ± SD.

If our printed raft domains maintain their native structure, we predicted that not only enzymes but also other membrane proteins should keep their biological function. We then characterized ligand binding to sigma-1 and sigma-2 receptors for their interest in neurodegenerative diseases. They are associated with AD^41^ and frontotemporal dementia and are considered targets for AD therapeutics.^42^ Our analysis of sigma receptors showed no statistical differences between conditions or cell types but instead a tendency toward less total ligand-binding to sigma-1 and sigma-2 receptors in neurons compared to astrocytes in the control situation (Figure 6A; 7727 ± 1864 and 11232 ± 3207 O.D/ng immobilized protein, respectively). Also, a tendency to decrease binding to astrocytic rafts upon stress conditions was observed (low-serum: 10353 ± 3389 O.D/ng immobilized protein; low-serum + paraquat: 7749 ± 2270 O.D/ng immobilized protein) (Figure 6A).

By using a sigma-1 masking agent, we analyzed sigma-2 receptor binding=. In this regard, binding activity presented differences between neuronal (3558 ± 953 O.D/ng immobilized protein) and astrocytic (7825 ± 549 O.D/ng immobilized protein) rafts in the control situation (*p-*value 0.003). Moreover, treatments in astrocytes also resulted in statistically significant differences between metabolic (6857 ± 950 O.D/ng immobilized protein) and oxidative (4617 ± 1815 O.D/ng immobilized protein) stress conditions (Figure 6B) (*p-*value of 0.0267). Finally, we evaluated the binding to sigma-1 receptors and obtained differences between cell types but not with conditions in astrocytes (Figure 6C). As expected from the behavior of sigma-2 receptor binding, our calculated sigma-1 binding has an opposite behavior to that obtained for total sigma receptor binding (5460 ± 1014 in neuronal rafts O.D/ng immobilized protein; 2295 ± 1890 O.D/ng immobilized protein in astrocytic rafts) (*p-*value 0.0582), whereas no differences are found for astrocytic rafts in different conditions (2320 ± 1997 O.D/ng immobilized protein in low serum starved rafts in the absence of paraquat and 2424 ± 2067 O.D/ng immobilized protein in its presence).

## Discussion

The present study develops a new methodology that allows for the simultaneous analysis of lipid composition, enzymatic activity, and receptor binding in raft domains isolated from neurons and astrocytes subjected to different stress conditions and immobilized in microarrays. To maintain the functionality of lipid raft proteins and make the isolation compatible with microarray printing, lipid raft isolation had to be optimized. It was first performed using two different detergents (TX-100 and TX-114) by incubating the cell suspension in buffer with the detergent at a final concentration of 1%. After raft separation in the sucrose gradient, a dialysis method was used to clean both the detergent and sucrose, which might affect the immobilization of the sample. Neither of these methods resulted in preparations that could be stably immobilized by our membrane-printing method. We therefore switched to a detergent-free method, followed by centrifugation steps to remove sucrose and reconstitute raft preparations in the printing buffer. Our results demonstrate the strength of this methodology in lipid fingerprint determination, cell type identification, and analysis of lipid and protein changes associated with exposure to metabolic or oxidative stress in raft microdomains.

To this aim, MALDI-MS experiments were performed by taking 20 laser shots/pixel, with 16 pixels per spot analyzed. Compared with MALDI-MS performed in individual samples, with this technology the time was significantly reduced, with a signal acquisition time of only 80 min per RMMA. With regard to lipid profiling, the results present a distinguishable lipid signature for raft subdomains not only between the different conditions (control, low serum starvation, and paraquat exposure) but also between different tissues (cell lines vs rat cortex homogenates)^30^ and even rafts from different brain cells (astrocytes vs neurons). As a relevant example, our data analysis revealed that [SM 34:2 – CH_3_]^−^ and [LPC 16:0 + Cs]^+^ are relatively enriched in astrocytic raft domains in every condition but is absent in neuronal rafts and in rat cortex (Figures 4B and S1B). This finding suggests that these lipids are present only in raft domains from human astrocytes, as the rat cortex internal control should contain both astrocytes and neurons. If that is the case, these species could be lipid biomarkers for the human astrocytic raft domains. Following the same reasoning, [PE 40:4 – H]^−^ could be a neuronal raft biomarker, as it is only detected in this domain (Figure 3B). In addition, various l+ipid variables were able to discriminate oxidative stress from metabolic stress and the last one from the control situation. As an example, [PA O-36:3 – H]^−^, observed only in metabolic and stressed situations, could be plasmalogen, which is related to protective functions against oxidative stress.^43^ One of the detected lipids, [PI 38:4 – H]^−^, with a higher presence in lipid rafts from the neuronal cell line compared to astrocytes (Figure 2B), is also found in white matter in human samples and is known to be downregulated in Alzheimer disease patients.^44^ In addition, [PE 36:1 – H]^−^, which is slightly increased in neuronal rafts (Figure 2B), has been described as a risk indicator for diabetes and was also found to be increased in HIV patients compared with the control situation.^45^ Therefore, the RMMA technology used to print raft domains provides a useful platform for comparative analysis of lipid signatures in a reasonable short time in order to determinate the potential effect over the lipidome of many toxic compounds such as paraquat or any treatment or disease where changes in lipid raft composition might be key to their understanding.

The maintenance of functionality of the lipid rafts printed in our RMMAs has been demonstrated thanks to the different enzymatic activity assays (NADH dehydrogenase, GAPDH, cholinesterase, acetylcholinesterase, and butyrilcholinesterase). Differences triggered by treatment in astrocytic rafts have been found in all enzymatic activities except in butyrilcholinesterase. In this sense, cholinesterase activity is known to be increased in activated astrocytes,^46^ explaining the differences between neuronal and astrocytic rafts. The differences between conditions in astrocytic raft domains can be produced by the recruitment and exclusion of proteins to and from the raft subdomains upon different stimuli. Therefore, the observed differences may indicate the existence of raft protein remodeling processes due to stress conditions or an alteration of the astrocyte functionality due to oxidative stress conditions.^47^ Revealing this fact is useful particularly for understanding AChE modulation due to its close relation with Alzheimer’s Disease (AD).^14^ Thus, our work opens the door to tests for a variety of treatments, such as anticholinesterase modulators or inhibitors, one of the main approaches for Alzheimer’s disease treatment. GAPDH is considered a housekeeping protein that has a great interest in several diseases such as AD^48^ or cancer.^49^ GAPDH showed a tendency toward increased activity in our printed raft domains due to paraquat treatment. Interestingly, paraquat treatment, seems to have an opposite effect at 48 h after treatment.^50^ Nevertheless, it has been reported that GAPDH increased activity or overexpression provides protection against apoptotic conditions.^51^

NADH is used as a substrate by different enzymes, including mitochondrial complex I, but not restricted to it. Thus, our results suggest that this component of the mitochondrial chain might be located in raft-like membrane subdomains.^52^ Also, it is important to notice that, as the signal in our enzymatic assays is normalized to the total protein present in each spot, the differences detected cannot be due to higher protein concentration in raft domains. It will be worth investigating if they are due to the lipid environment influences on enzyme activity.

The RMMA methodology has also proved to be useful for receptor binding assays. We have tested sigma-1 and sigma-2 binding and found higher receptor density in rafts from astrocytes compared to neurons, with a decreasing tendency in metabolic and oxidative stress conditions. Using a sigma-1 masking agent (see material and methods section) we can detect that statistically significant differences were present between metabolically and oxidative stressed raft domains (Figure 6C). No differences were observed between conditions for sigma-1 receptor binding in astrocytic rafts. Curiously, sigma-2 receptors have been found in brain regions and are closely associated with lipid metabolism, amyloid-β oligomer blocking, synaptoprotection, and regulation of cholesterol homeostasis among other functions.^53^ Thus, the aberrant activity of processes that are sigma-2-mediated can be triggered by oxidative stress. Nevertheless, this pathological process can also alter the membrane composition and promote their exclusion from rafts. Last but not least, the potential of this methodology is not limited to these techniques but also to immunoassays, proteomic analysis, or drug screening analysis.

## Conclusion

We present here an improved microarray methodology to analyze the lipidome, protein enzymatic activity, and receptor density in raft membrane domains immobilized in microarrays. As a summary, this technology allows the performance of several techniques, with special attention to the MALDI-MS protocol in raft domains using smaller sample amounts and less time than standard mass spectrometry techniques. Furthermore, RMMA technology allows the possibility of performing several techniques in a variety of tissues and samples, using the same initial samples, and analyzing covariance between outcomes that might reveal interesting physiological processes.

## Supporting Information Available

List of drugs and reagents, additional information on Western blot performance, additional figures for lipid spectra extracted from RMMAs in negative and positive ion mode, relative abundance and segmentation analysis in positive ion mode, tables with lipid species annotation, principal component analysis of positive ion mode data, table of classification of samples using different classification algorithms (PDF)
