# Supplementary material for: Microarray-Based Methodology for Lipid Profiling, Enzymatic Activity, And Binding Assays in Printed Lipid Raft Membranes from Astrocytes and Neurons
Source: Anal Chem. 2024 Dec 24;97(1):86–95. doi: 10.1021/acs.analchem.4c02421 (PMC11740170; doi:10.1021/acs.analchem.4c02421)
Supplement: Supplementary file 1 — ac4c02421_si_001.pdf [file ac4c02421_si_001.pdf]

SUPPORTING INFORMATION

Microarray-based methodology for lipid profiling, enzymatic activity and binding assays in printed lipid rafts membranes from astrocytes and neurons

Laura Sánchez-Sánchez[1,2,*], Roberto Fernández[1], Egoitz Astigarraga[1], Gabriel Barreda-Gómez[1#], María Dolores Ganfornina [2 #].

[1] IMG Pharma Biotech S.L, Zamudio, 48170, Spain
[2] Instituto de Biomedicina y Genética Molecular, Unidad de Excelencia, University of Valladolid-CSIC, Valladolid, 47003, Spain
# Equal contribution. * Corresponding author

***Corresponding author information**

Dr. Laura Sanchez-Sanchez
Phone: +1 4094579250
Email: laura.sanchez@estudiantes.uva.es / lauusnchzbna@gmail.com
ORCID: 0000-0001-5777-6588

Table of contents

Drug and reagents S2

Additional information on western blot performance S2

Table S1: Assignation of noted lipids, and its experimental m/z....S2

Table S2: Classification of astrocytic rafts in oxidative *versus* metabolic stress using 50-best ranked noted lipids....S3

Table S3: Classification of astrocytic rafts in metabolic stress *versus* control situation using 50-best ranked noted lipids....S3

Figure S1: Lipid fingerprint analysis in RMMAs from astrocytic and neuronal cell lines using mass spectrometry in MS+. A) Printing design for microarrays. Standard curve and samples printed in triplicates B) Example images showing the relative abundance of selected lipids in raft samples or standards using gray scale. C) Segmentation analysis using our modified RankCompete algorithm. Colors were assigned using the rainbow color scale. Clusters that include rat cortex were removed. S4

Figure S2: Principal component analysis (PCA) of lipid raft samples from neuronal and astrocytic cell lines in different conditions in using MS^+ data. A) PCA of astrocytic and neuronal rafts in control situations (99% of variability explained by PC1 and PC2). B) PCA of astrocytic rafts in metabolic stress and control situation (98% of variability explained by PC1 and PC2). C) PCA of astrocytic rafts in metabolic and oxidative stress (91% of variability explained by PC1 to PC4). S4

Figure S3: Lipid spectra extracted from RMMAs. A) Negative ion mode spectra from neuronal and astrocytic rafts in different conditions. B) Positive ion mode spectra from neuronal and astrocytic rafts in different conditions. S5

Drug and reagents

CELT-483 fluorescent ligand, and L6 sigma 1 masking agent were purchased at Celtarys Research (Galiza, Spain). Nitrotetrazolium blue chloride (NBT), beta-nicotinamide adenine dinucleotide (NADH), decylubiquinone (dUQ), sodium azide, cytochrome c from equine heart, Trizma base, dimetil sulfoxide, sodium deoxycholate, Tween 20, bovine serum albumin (BSA), copper sulfate, sodium citrate tribasic dihydrate, trizma maleate, acetylthiocholine iodide, 1,5 Bis(4-Allydimethylammoniumphenyl)5-3-1-2Br (BW284), Triton X-100, Methyl viologen dichloride hydrate (Paraquat), protease inhibitor cocktail, sucrose, were purchased from Sigma-Aldrich (Saint Louis, MO, USA). Potassium hexacyanoferrate (III), acrylamide-bis ready-to-use solution 30%, APS 10%, chlorhidric acid (HCl) 37%, TEMED, were purchased from Merck (Darmstadt, Germany). Methanol was purchased from Panreac (Catalonia, Spain). Cessium chloride was purchased from HoneyWell (Charlotte, NC, USA). Dithiothreitol (DTT) was purchased from Bio-Rad laboratories (Hercules, CA, USA). Dodecyl sulfate sodium salt, 99% for biochemistry was purchased from Acros Organics, EDTA tetrasodium tetrahydrate salt was purchased from Calbiochem (Geel, Belgium). Enhanced chemiluminescencereagents were purchased from Merck-Millipore. Trichloroacetic acid (TCA) was purchased from Carlo Erba Reagents (Cornaredo,Italy). Micro BCA protein assay kit was purchased from ThermoFisher Scientific (Waltham, MS, USA).

Additional information on western blot performance

Trichloroacetic acid protein precipitation: proteins were precipitated to be resuspend in small volumes. Trichloroacetic acid (TCA) was added to each fraction from a 100% stock to get a final 20% concentration. After vortexing and incubation at 4ºC for 20 minutes, samples are centrifuged (30 minutes, 16,100 g, 4ºC, 5415R centrifuge, Eppendorf, Hamburg, Germany). Supernatants are removed, absolute ethanol (1 ml at -20ºC) is added, and samples are mixed by inversion. This ethanol wash is repeated three times. After discarding the last supernatant pellets are dried at 35 ºC for 15 minutes (SPD Speed Vac, SPDmV, ThermoFisher Sci., Waltham, MA, USA) and stored at -20ºC until usage.

Western blot assay. For western blot assays, protein extracts were mixed with protein sample buffer adapted for TCA-precipitated samples (63 mM Tris-HCl, 10% glycerol, 2% SDS, 100 mM DTT, 0.05% bromophenol blue) and heated at 70 ºC for 5 minutes. Discontinuous SDS-PAGE electrophoresis (acrylamide 12% in resolving gel; 3% in stacking gel) was used, and 15 µg of total protein was loaded per lane. Electrophoresis was performed at constant 80 V through the stacking gel and 120 V through the resolving gel for approximately 2 hours. PVDF membranes pre-activated with methanol for 15 seconds and washed with Transfer buffer (25 mM Tris, 190 mM Glycine, 20% MetOH) were used for blotting. Proteins were then transferred from the gel to the membranes at constant 400 mA current for 1.5 hours in a Western blot system (BioRad Laboratories, Hercules, CA, USA) with immersion in Transfer buffer. PVDF membranes were washed in TBS-Tw solution (50 mM Tris, pH 7.5, 150 mM NaCl, 0.05% Tween-20), blocked in 5% of powdered milk in TBS-Tw buffer, and incubated with rabbit serum anti-hApoD 0.2 µg/ml (1:1000 dilution, custom made, 9), mouse anti-caveolin-1 0.2 µg/ml (1:1000 dilution, ref sc-894; Santa Cruz Biotechnology), mouse anti-Lamp2 0.26 µg/ml (1:500 dilution, H4B4 monoclonal, DSHB), or mouse anti-Flotillin-1 0.25 µg/ml (1:1000 dilution, ref 610820, Becton Dickinson) for 2 hours at room temperature or overnight at 4ºC. After four 10-minute washes with TBS-Tw, membranes were incubated with secondary antibody (goat anti-mouse IgG-HRP, 1:10000 dilution, Dako) in blocking solution for 1 hour at room temperature. After washing as described above, the presence of the different markers was revealed using Enhanced Chemiluminescence (ECL, Pierce). The signal was visualized with Versadoc Molecular Imager coupled with a high-sensitivity CCD camera (Versadoc Imaging system 5000, Bio-Rad Laboratories, Hercules, CA, USA). The integrated optical density of the immunoreactive protein bands was measured in images taken within the linear range of the camera, avoiding signal saturation. To detect different proteins in the same blot, PVDF membranes were subject to two 10 minutes incubations with stripping buffer (3.5 mM SDS, 1% Tween-20, 0.2 M glycine, 0.1 N HCl) and then washed 5 times with TBS-Tw solution.

Table S1: Assignation of noted lipids, and its experimental m/z

Experimental m/z	Theorical m/z	Annotation	Delta
**885.5524**	885.5499	$[PI\ 38:4-H]^-$	0.0025
**794.5730**	794.5705	$[PE\ 40:4-H]^-$	0.0025
**788.5451**	788.5447	$[PS\ 36:1-H]^-$	0.0004
**760.5164**	760.5134	$[PS\ 34:1-H]^-$	0.003

**744.5581**	744.5549	[PE 36:1-H]$^-$	0.0033
**685.5294**	685.5290	[SM 34:2-CH_3]$^-$	0.0004
**659.5117**	659.5134	[SM 32:1-CH_3]$^-$	0.0017
**655.4703**	655.4708	[PA O-34:3-H]$^-$	0.0005
**599.3227**	599.3202	[LPI 18:0-H]$^-$	0.0025
**892.4810**	892.4832	[PC 34:1+Cs]$^+$	0.0022
**904.4824**	904.4823	[PC 35:2+Cs]$^+$	0.0001
**924.4506**	924.4510	[PC 37:6+Cs]$^+$	0.0004
**628.2350**	628.2370	[LPC 16:0+Cs]$^+$	0.0020

Table S2: Classification of astrocytic rafts in oxidative *versus* metabolic stress using 50-best ranked noted lipids

Model	*AUC*	*CA*	*F-1*	*PR*	*Recall*
kNN	1.0	1.0	1.0	1.0	1.0
Random Forest	1.0	1.0	0.917	0.917	0.917
Neural network	1.0	1.0	1.0	1.0	1.0
Naïve Bayes	1.0	1.0	1.0	1.0	1.0

Table S3: Classification of astrocytic rafts in metabolic stress *versus* control situation using 50-best ranked noted lipids

Model	*AUC*	*CA*	*F-1*	*PR*	*Recall*
kNN	1.0	1.0	1.0	**1.0**	**1.0**
Random Forest	1.0	1.0	1.0	1.0	1.0
Neural network	1.0	1.0	1.0	1.0	1.0
Naïve Bayes	1.0	1.0	1.0	1.0	1.0

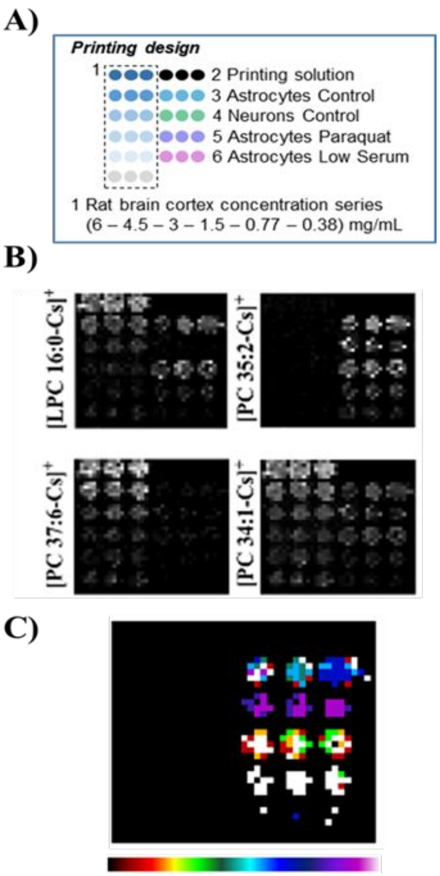

Figure S1: Lipid fingerprint analysis in RMMAs from astrocytic and neuronal cell lines using mass spectrometry in MS+. A) Printing design for microarrays. Standard curve and samples printed in triplicates B) Example images showing the relative abundance of selected lipids in raft samples or standards using gray scale. C) Segmentation analysis using our modified RankCompete algorithm. Colors were assigned using the rainbow color scale. Clusters that include rat cortex were removed.

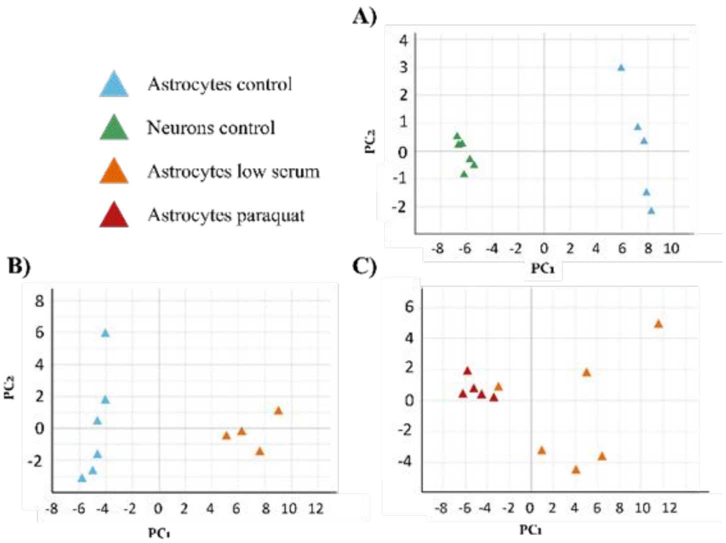

Figure S2: Principal component analysis (PCA) of lipid raft samples from neuronal and astrocytic cell lines in different conditions in using MS+ data. A) PCA of astrocytic and neuronal rafts in control situations (99% of variability explained by PC1 and PC2). B) PCA of astrocytic rafts in metabolic stress and control situation (98% of variability explained by PC1 and PC2). C) PCA of astrocytic rafts in metabolic and oxidative stress (91% of variability explained by PC1 to PC4).

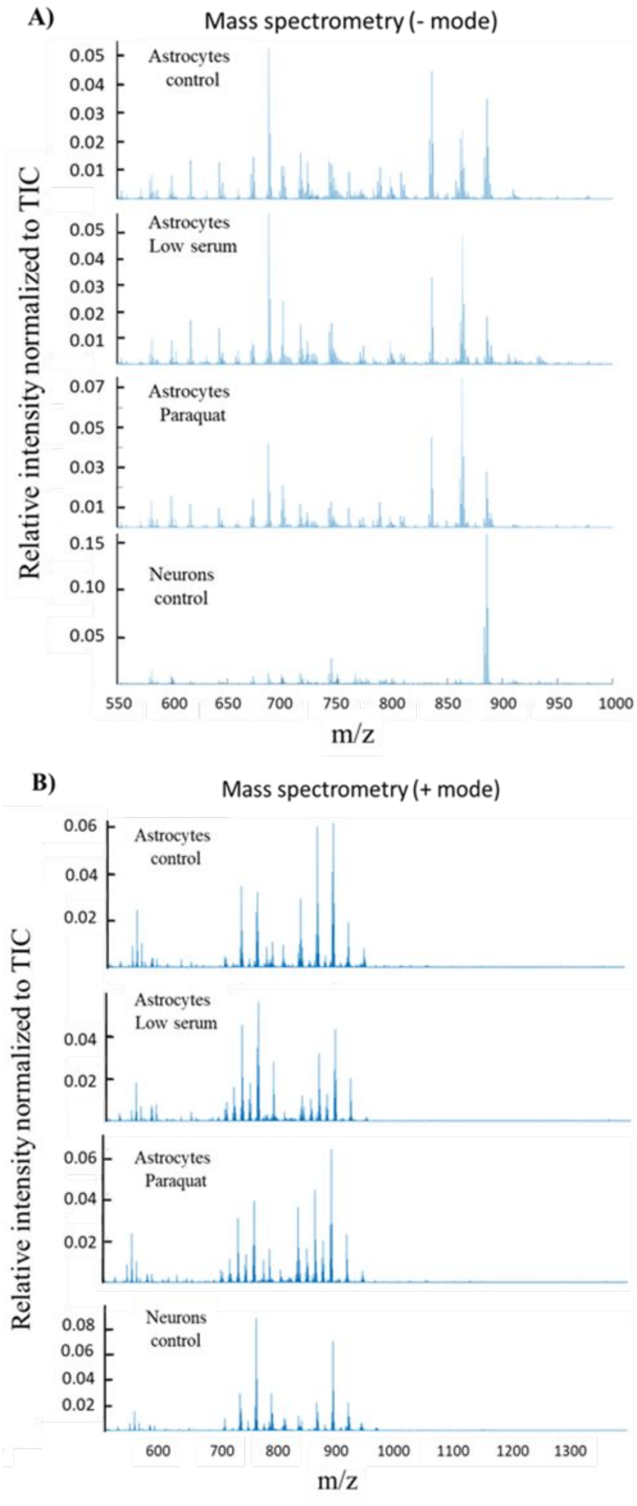

Figure S3: Lipid spectra extracted from RMMAs. A) Negative ion mode spectra from neuronal and astrocytic rafts in different conditions. B) Positive ion mode spectra from neuronal and astrocytic rafts in different conditions.
